# Hepatitis B prevalence and risk factors among adults living with HIV in South Africa: a clinic-based cohort study

**DOI:** 10.1186/s12879-024-09746-7

**Published:** 2024-08-31

**Authors:** Megana Shivakumar, Caitlin A. Moe, Ashley Bardon, Meighan Krows, Sabina Govere, Mahomed Yunus S. Moosa, Connie Celum, Paul K. Drain

**Affiliations:** 1 Department of Global Health, University of Washington, 908 Jefferson St., 12th floor, Seattle, WA 98104 USA; 2 Department of Epidemiology, University of Washington, Seattle, USA; 3 Global Health Center, Washington University, St. Louis, USA; 4 AIDS Healthcare Foundation, Durban, South Africa; 5 Department of Infectious Diseases, University of KwaZulu-Natal, Durban, South Africa; 6 Department of Medicine, University of Washington, Seattle, USA

**Keywords:** HIV, HBV, Co-infection, Risk factors

## Abstract

**Background:**

People living with HIV (PLHIV) may have concurrent Hepatitis B Virus (HBV) infection, and certain antiretroviral therapies are recommended for HBV-HIV co-infected individuals. Routine screening for Hepatitis B virus may influence management of antiretroviral therapy for PLHIV, but risk factors for co-infection have not been well defined. The objective of this study was to identify risk factors for HBV infection among PLHIV in South Africa.

**Methods:**

We conducted a cross-sectional analysis of a prospective, clinic-based cohort study of adults seeking HIV testing from 2013–2017 in Umlazi township, South Africa. Patients newly diagnosed with HIV were enrolled and subsequently tested for Hepatitis B surface antigen positive (HBsAg +). We used a Poisson linear regression model to assess which factors, pertaining to sociodemographic status, medical history, clinical symptoms, mental health were associated with HBV.

**Results:**

Among 3,105 PLHIV participants in South Africa, 6% were positive for HBV. Males had a higher HBV prevalence (10.4%) than females (5.2%). Within the HBV-positive group, the mean age was 33.2 years, with 38.3% females and 43.9% having completed high school or higher. About 39.9% reported alcohol use, 24.7% had a smoking history, and 8.3% reported substance use in the past year. Older participants born before 1995, when routine infant HBV vaccination was introduced, were more likely to have HBV. In multivariable analyses, smoking history increased HBV risk in females (aPR = 2.58; 95% CI 1.47–2.52), while alcohol use decreased HBV risk in males (aPR = 0.36; 95% CI 0.19–0.70).

**Conclusions:**

In a South African cohort, roughly one in 16 PLHIV had HBV co-infection, and this rate was higher in males. The most prominent risk factors for HBV infection in PLHIV were alcohol use, higher income, and smoking history, which may help inform targeted treatment and prevention strategies. Creating HBV-specific screening and prevention strategies for PLHIV may be useful for reducing HBV infections.

## Introduction

Human Immunodeficiency Virus (HIV) and Hepatitis B Virus (HBV) are both major causes of global morbidity and mortality. Worldwide, roughly 10% of people living with HIV (PLHIV) may have concurrent HBV infection [1–3]. In South Africa, HIV and HBV prevalence are 10% and 8–13%, respectively, although HIV incidence varies regionally [4–6]. Within the KwaZulu-Natal province of South Africa, where this study was conducted, HIV prevalence is estimated as 20.7% [7]. Since the two infections share similar transmission patterns, understanding factors associated with their co-infection may improve diagnosis and outcomes.

Identifying risk factors for HIV and HBV co–infection can be important for directing therapy and reducing transmission of both viruses. In addition, assessing HBV co–infections among PLWHIV can facilitate optimal anti-viral medications to treat both infections. Several nucleoside reverse transcriptase inhibitors used to treat HIV, such as tenofovir and lamivudine, are effective for the treatment of HBV [8]. The main common risk factors for HIV and HBV are the same due to the similar transmission methods, including lack of condom use and injection drug use [9, 10]. In this study, we sought to identify factors associated with HBV infection among patients recently diagnosed with HIV in South Africa.

## Methods

### Study setting and population

We conducted a cross-sectional analysis of HBV prevalence among adults testing for HIV at the iThembalabantu clinic between September 2013 to February 2017 in KwaZulu–Natal, South Africa. Study participants were recruited from the outpatient department of the iThembalabantu Clinic and enrolled in the cohort study before they were tested by a trained counselor for HIV. Informed consent to participate was obtained from all of the participants in the study. The iThembalabantu Clinic is an outpatient HIV clinic located in the Umlazi Township, a highly HIV-endemic area in the Province of KwaZulu-Natal, South Africa [11]. This clinic provides comprehensive testing, treatment, and care to PLHIV.

Study participants were aged ≥ 18 years, with unknown HIV status at time of recruitment, ART naïve status, and seeking HIV testing at the iThembalabantu Clinic. HBV testing was only available to persons who were confirmed HIV positive by the clinic providers. Individuals were excluded if they were pregnant or had received anti-fungal therapy within 3 months of enrollment into this study. All enrolled patients provided written, informed consent in either English or isiZulu.

After enrollment into this study, but before HIV testing, a research assistant collected information about socio-demographic characteristics, economic and behavioral factors, HIV testing history, and the participant’s perception of their risk of acquiring HIV. These questions were asked before HIV testing, as some patient answers may be influenced by a new diagnosis of HIV [12]. Participants were provided a clinical examination and assessed for any signs or symptoms of other and laboratory testing, including CD4 count, HIV viral load, and HBV testing.

### Measures

The outcome of interest for this study was the presence or absence of HBV infection at the time of HIV diagnosis. HBV cases were defined as individuals who were Hepatitis B surface antigen positive (HBsAg +) using a blood antibody test. Laboratory testing was performed at the National Health Laboratory Service at Prince Myshenyi Memorial Hospital in Umlazi.

Potential risk factors were identified and selected based on a review of the literature about potential risk factors for HBV and HIV infections. Factors examined included: sex, alcohol use over the past year, smoking history, substance use, contraception use, and depression. Smoking history was measured as ever reporting use of cigarettes. Substance use was measured as ever reporting use of any of the following: intravenous drug use, whoonga, cannabis, methamphetamine, hallucinogens, glue, or ecstasy. Contraception use was measured as ever using any form of contraception. Depression was measured through a series of questions that were created using the DSM–5. All risk information about risk factors were asked prior to HIV testing. Participants with incomplete results were removed prior to analysis.

Risk factors examined included sex, alcohol use over the past year, smoking history, substance use, contraceptive use, and depression. Alcohol consumption was chosen as increased consumption can weaken the liver, allowing HBV to persist chronically [13]. Contraceptive use was measured as HBV can be acquired via sexual contact. Any substance use was evaluated as a known risk factor for HBV infection among PLHIV who use IV drugs [14]. The risk factors of anxiety and depression were chosen as there is increased prevalence of both in PLHIV, so they may contribute to risk behaviors that increase the risk of acquisition of HBV [15].

### Statistical analyses

The multivariable models were adjusted for age (in years), employment status, income indicator, high school or greater educational attainment, and marital status. We used chi-squared tests to compare sample characteristics by sex. We fit Poisson linear regression models with log links and robust standard errors to examine associations between specified risk factors and HBV status. We separately evaluated those born before and after 1995, since South Africa implemented routine infant vaccination for HBV in 1995. Due to the notable sex differences within this cohort, we included an interaction term to allow associations between each risk factor and HBV status to vary by sex. Statistical calculations were performed in Stata version 17 (StataCorp, College Station, TX).

### Ethical review process

Ethical approval was gained from the University of Washington’s Institutional Review Board #49563 and from the Biomedical Research Ethics Committee of the University of KwaZulu-Natal, reference BF052/13. This study conforms to STROBE reporting guidelines.

## Results

In this clinic-based cohort, 3,105 PLHIV were enrolled from September 2013 to February 2017 and included in this study. Among those, 196 (6.3%) also tested positive for HBV. Among all participants, the mean age was 33.2 years, 1,774 (57.1%) were female, 1,525 (49.1%) completed high school or higher, and 1,312 (42.3%) were employed (Table 1). Among participants who tested positive for HBV at baseline, the mean age was 33.2, 75 (38.3%) were female, 86 (43.9%) completed high school or higher, and 92 (46.9%) were employed (Table 1). Overall, 1,239 (39.9%) participants reported using alcohol over the past year, 767 (24.7%) participants reported any smoking history, and 258 (8.3%) participants reported any substance use.

**Table 1:** Sample characteristics, *N* = 3105

	**HBV-Positive,***N* = 196	**HBV-Negative,***N* = 2422	**Unknown HBV Status,***N* = 466	**Overall, ***N* = 3105
	n (%)			
Age (years): mean (SD)	33.2 (7.5)	33.3 (9.4)	32.8 (9.4)	33.2 (9.3)
Female sex	75 (38.3)	1380 (57.0)	319 (65.5)	1774 (57.1)
Marital status				
Married	6 (3.1)	160 (6.6)	27 (5.5)	193 (6.2)
Single (never married)	189 (96.4)	2233 (92.2)	457 (93.8)	2879 (92.7)
Widowed/Divorced	1 (0.5)	29 (1.2)	3 (0.6)	33 (1.1)
Employed	92 (46.9)	1002 (41.4)	218 (44.8)	1312 (42.3)
Contraception use (any form) ^(b)^	13 (6.6)	259 (10.7)	51 (10.5)	323 (10.4)
Income > 10,000 ZAR/month ^(c)^	6 (3.1)	42 (1.7)	2 (0.4)	50 (1.6)
High school education or higher	86 (43.9)	1232 (50.9)	207 (42.5)	1525 (49.1)
Previously received HIV testing	147 (75.0)	1876 (77.5)	367 (75.4)	2390 (77.0)
Partner HIV status ^(d)^				
HIV +	59 (30.1)	632 (26.1)	156 (32.0)	847 (27.3)
HIV -	24 (12.2)	403 (16.6)	81 (16.6)	508 (16.4)
Unknown	113 (57.7)	1369 (56.5)	250 (51.3)	1732 (55.8)
Depression (mild or worse) ^(e)^	31 (16.1)	374 (15.6)	178 (37.1)	583 (19.0)
Substance use (ever) ^(f)^	16 (8.2)	219 (9.1)	23 (4.7)	258 (8.3)
Cigarette use (ever) ^(g)^	68 (34.7)	571 (23.6)	128 (26.4)	767 (24.7)
Alcohol use past year ^(a)^	95 (48.5)	972 (40.1)	172 (35.3)	1239 (39.9)

^(a)^missing *n* = 8

^(b)^missing *n* = 160

^(c)^missing *n* = 27

^(d)^missing *n* = 18

^(e)^missing *n* = 37

^(f)^missing *n* = 20

^(g)^missing *n* = 5

**Fig. 1:**
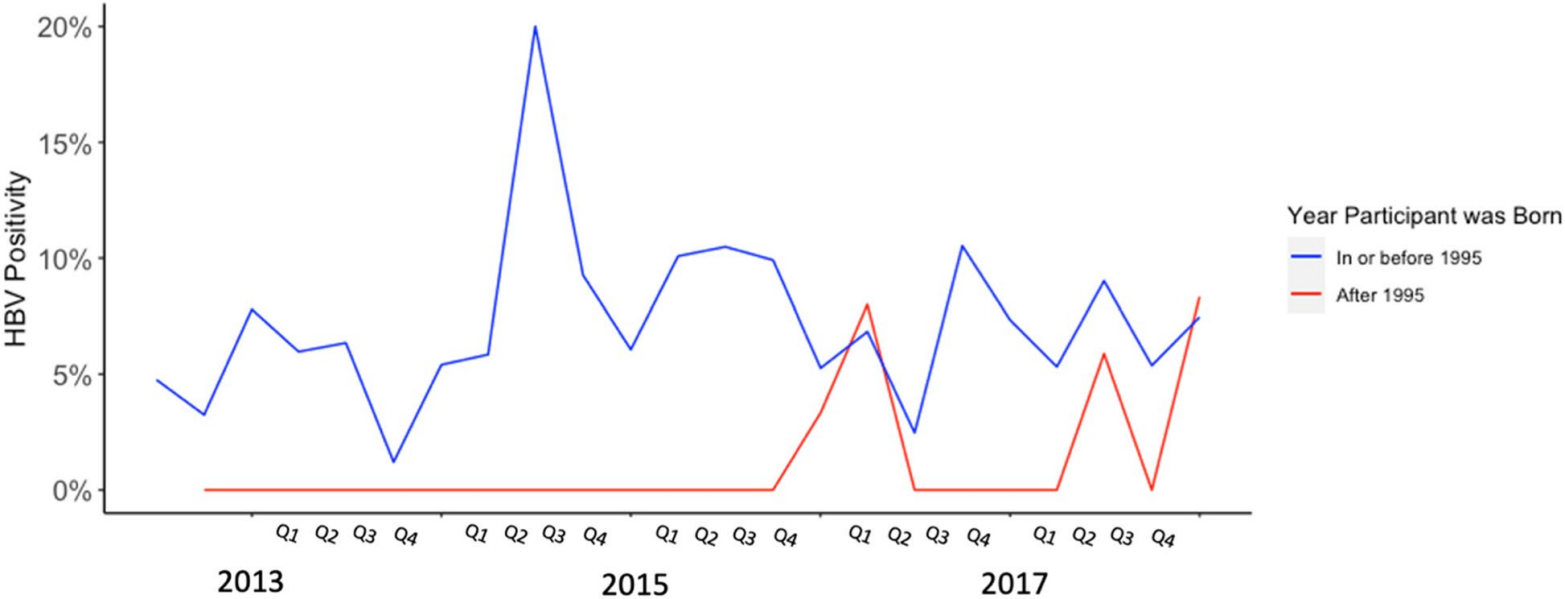
HBV prevalence based on participant birth year

In this cohort, 2958 participants (95.0%) were born before 1995, before South Africa implemented routine infant HBV vaccination. Compared to participants born after 1995, and ostensibly received HBV vaccinations, older participants were more likely to have HBV (*p* = 0.043). Among participants who were born before 1995, females were less likely to have HBV compared to males (*p* < 0.001). The point prevalence of HBV among our cohort over the course of enrollment, among those born before versus after 1995 is shown in Fig. 1.

There were statistically significant differences in HBV positivity by sex. Males (10.4%) were more likely than females (5.2%; pairwise *p* < 0.001) to have HIV/HBV co–infection (Table 2). There were also statistically significant differences by sex in alcohol use over the past year and income, in that men were more likely to report using alcohol over the past year and an income > 2,000 ZAR/month. There were no statistically significant differences with educational attainment beyond high school, married marital status, or depression symptoms by sex.

**Table 2:** Characteristics of participants by sex, *N* = 3105 newly diagnosed PLHIV

	**Male (*N* = 1331)**	**Female (*N* = 1774)**	***P*-value**
	n (%)		
HBV-HIV Co-infection	121 (10.4)	75 (5.2)	< 0.001
Currently employed	704 (52.9)	608 (34.3)	< 0.001
Income > 2,000 ZAR/month	501 (38.0)	351 (20.0)	< 0.001
High school education or higher	629 (47.3)	896 (50.5)	0.073
Cigarette smoker	626 (47.2)	141 (8.0)	< 0.001
Alcohol use past year	757 (57.1)	482 (27.2)	< 0.001
Substance use^a^	205 (15.5)	53 (3.0)	< 0.001
Contraception use	14 (1.2)	309 (17.6)	< 0.001
Never married	1237 (92.9)	1642 (92.6)	0.688
Depression	123 (9.3)	176 (10.1)	0.510

^a^Includes reported use of any of the following: intravenous drug use, whoonga, cannabis, methamphetamine, hallucinogens, glue, or ecstasy

In multivariable analyses among females, those who reported any smoking history were 2.58 times more likely to test positive for HBV (aPR = 2.58; 95% CI 1.47–2.52) compared to females with no smoking history (Table 3). In multivariable analyses among males, those who reported alcohol use in the past year were 64% less likely to test positive for HBV, compared to males who did not report past year alcohol use (aPR = 0.36; 95% CI 0.19–0.70) (Table 3). In both adjusted and unadjusted analyses, contraception use among males was associated with a statistically significantly increased likelihood of testing positive for HBV (aPR < 0.01; 95% CI < 0.001–0.01).

**Table 3:** Associations between specified risk factors and HBV-HIV co-infection among a cohort of newly diagnosed PLHIV, *N* = 31,058/13/2024 9:59:00 AM

	Unadjusted Models				Adjusted Models			
	**Males**		**Females**		**Males**		**Females**	
	**PR (95% CI)**	***P*-value**	**PR (95% CI)**	***P*-value**	**aPR (95% CI)**	***P*-value**	**aPR (95% CI)**	***P*-value**
Alcohol use past year	0.57 (0.32, 0.99)	0.049	1.59 (1.01, 2.51)	0.045	0.57 (0.32, 0.99)	0.048	1.55 (0.99, 2.44)	0.054
Cigarette smoker	0.35 (0.18, 0.67)	0.002	2.78 (1.58, 4.88)	< 0.001	0.36 (0.19, 0.70)	0.002	2.58 (1.47, 4.52)	0.001
Substance use	0.73 (0.17, 3.20)	0.681	0.90 (0.23, 3.55)	0.880	0.75 (0.17, 3.35)	0.711	0.82 (0.20, 3.33)	0.783
Contraception use	< 0.001 (< 0.001, 0.001)	< 0.001	0.95 (0.53, 1.70)	0.862	< 0.001 (< 0.001, 0.001)	< 0.001	0.91 (0.51, 1.63)	0.759
Depression	1.07 (0.41, 2.79)	0.884	1.16 (0.55, 2.48)	0.694	1.08 (0.42, 2.81)	0.874	1.13 (0.52, 2.41)	0.761

Models adjusted for: age (in years), employment status, income indicator, high school or greater educational attainment, and marital status

Statistical methods: we used chi-squared tests to compare sample characteristics by sex. We fit Poisson linear regression models with log links and robust standard errors to examine associations between specified risk factors and HBV status. Due to the notable sex differences in this cohort, we included an interaction term to allow associations between each risk factor and HBV status to vary by sex

In other words, unadjusted models took the form:

$$\text{log}\left({\lambda }_{i}\right)= {\beta }_{0}+ {\beta }_{1}{x}_{1}+ {\beta }_{2}sex+\gamma {x}_{1}*sex$$

Where ${\lambda }_{i}$ is the outcome, HBV positivity, and ${x}_{1}$ is the risk factor of interest

The fully adjusted models accordingly were fit as follows:

$$\text{log}\left({\lambda }_{i}\right)= {\beta }_{0}+ {\beta }_{1}{x}_{1}+ {\beta }_{2}sex+\gamma {x}_{1}*sex+ {\beta }_{3}{{\varvec{X}}}_{i}$$

Where in addition to ${\lambda }_{i}$ signifying the HBV outcome and ${x}_{1}$ representing the risk factor of interest, ${{\varvec{X}}}_{i}$ represents a vector of sociodemographic covariates: age (in years), employment status, income indicator, high school or greater educational attainment, and marital status

PR Prevalence ratio, aPR Adjusted prevalence ratio, CI Confidence interval

## Discussion

Among a cohort of 3,105 adults in Umlazi township, South Africa, roughly one in 16 PLHIV had HBV co-infection. HIV/HBV co–infection was found to be more common in males compared to females. The main risk factor found for HIV/HBV co–infection among females was smoking history and alcohol use in the past year. Among males, past-year alcohol use and smoking history were associated with a decreased risk of HBV co-infection.

Due to the similar transmission methods for HIV and HBV, many of the risk factors for each individual disease is shared for HIV/HBV co–infections [16]. Some differences between childhood transmission for HBV and HIV are present, as HIV is congenital in nature. In addition, due to the increased risk of liver failure from HBV and immune system dysfunction due to HIV, individuals with co–infection are at higher risk of chronic progression [16]. PLHIV with a suppressed immune system may experience worse symptoms when infected with HBV compared to those with an HBV mono–infection [17]. Previous studies have been conducted looking at sex differences within HBV infection, where men are more prone to HBV due to the varying effects of sex hormones [18].

The association of lack of barrier contraception use as a risk factor for HIV and HBV co–infection has been found in other studies, though few other studies evaluated HBV co–infection among PLHIV [19, 20]. A study of individuals to compare HBV prevalence in Roma to non–Roma populations found that the infrequent use of condoms was a significant risk factor for anti-Hepatitis B antibody positivity [21]. The study found that the main method of horizontal transmission in the Roma and non–Roma population was likely sexual intercourse. In a study on HBV conducted in Peru, a similar conclusion of condom usage being associated with a lower prevalence of Hepatitis B core antibodies was found after adjusting for gender, geographic region, age at sexual debut, year of study, education level, and lifetime number of sexual partners [22]. In another study of individuals age 40–49 years old with HBV, increased condom usage was shown to greatly decrease the prevalence of HBV infections [23]. In addition, it was found that the regular use of condoms could reduce the prevalence of anti HBV antibodies three-fold [22]. These studies showed how the lack of consistent condom usage may be a risk factor for HBV and HIV co–infection, which was supported by the findings in our cohort of PLHIV.

Alcohol consumption may be another important risk factor, and alcohol use is more common in men compared to women [24, 25]. Additionally, smoking and being male both increase the risk of higher alcohol consumption, both of which are risk factors shown for HBV [26]. These risk factors may be attributed to social and cultural factors, as there has historically been a greater acceptance of alcohol consumption among males. This could lead to higher levels of alcohol consumption among men as they may feel more social pressure to conform to these norms. In our analyses, both alcohol consumption and smoking were risk factors for HBV infection. Overall, a combination of social, cultural, and behavioral factors, coupled with lower vaccination rates, may have significantly increased the risk of HBV infection among older men.

Developing a specific prevention strategy to reduce the chance of contracting HBV may be valuable for PLHIV [27]. Although pre–exposure prophylaxis is a preventative medication for HIV, the most effective method to prevent individuals from contracting HBV is vaccination [28]. Once individuals are diagnosed with both HIV and HBV, highly active antiretroviral therapies can be used to treat both HIV and HBV. The three most common medications prescribed are lamivudine (3TC), tenofovir (TDF), and emtricitabine (FTC) [29]. These medications are effective against both HIV and HBV [30]. Patients who have HIV and are later exposed to HBV may experience rapid progression of HBV disease towards cirrhosis and hepatocellular carcinoma compared to mono-infection with HBV, as co–infection is associated with a more aggressive disease course of HBV [31]. Sudden discontinuation of treatment can be dangerous as it can lead to hepatic flares and liver failure as a result of the HBV infection [32].

This study has some strengths and limitations. Our findings are representative of the larger population of PLHIV in the region, but assessment was restricted to one large HIV clinic. Responses to questionnaires might be subject to recall bias. However, strengths of this study include the large sample of PLHIV and test naïve status of all participants. Finally, our findings may be attributable to unmeasured confounders, reverse causality, or chance findings due to multiple comparisons.

In conclusion, the main risk factors for HIV and HBV co-infection found from this study were alcohol use over the past year, and having a high income. For PLHIV who exhibit any of the known risk factors, counseling from health care providers about preventative medication or reducing activities that may increase their chance of developing HBV and building support systems may be useful. Finally, the differential age risk associated with HBV suggests that the introduction of HBV vaccination has had increased protection to HBV infection among PLHIV in South Africa. The younger group also has less HBV exposure and decreased prevalence, suggesting an important role for HBV vaccination programs. Overall, these findings can inform treatment and prevention programs for HBV-HIV co-infected individuals.

## Data Availability

Data can be provided upon reasonable request.

## Abbreviations

PLHIV: People living with HIV
HBV: Hepatitis B Virus
HBsAg +: Hepatitis B surface antigen positive
HIV: Human Immunodeficiency Virus
3TC: Lamivudine
TOF: Tenofovir
FTC: Emtricitabine
